# Increased ICU mortality in septic shock patients with hypo- or hyper- serum osmolarity: A retrospective study

**DOI:** 10.3389/fmed.2023.1083769

**Published:** 2023-02-01

**Authors:** Gang Heng, Jiasi Zhang, Yi Dong, Jiankun Jia, Benqi Huang, Yanbing Shen, Dan Wang, Zhen Lan, Jianxin Zhang, Tao Fu, Weidong Jin

**Affiliations:** ^1^Department of General Surgery, PLA Middle Military Command General Hospital, Wuhan, China; ^2^Center of Haematology, Southwest Hospital, Army Medical University, Chongqing, China; ^3^The First School of Clinical Medicine, Southern Medical University, Guangzhou, China; ^4^Department of Quality Education, Jiangsu Vocational College of Electronics and Information, Huaian, China

**Keywords:** septic shock, serum osmolarity, hyper-osmolarity, hypo-osmolarity, ICU mortality

## Abstract

**Background:**

While many factors that are associated with increased mortality in septic shock patients have been identified, the effects of serum osmolarity on the outcomes of ICU patients with septic shock have not yet been studied.

**Methods:**

The present study was designed to examine the association of serum osmolarity with ICU 28-day mortality in ICU patients with septic shock. Adult patients diagnosed with septic shock from the MIMIC-IV database were selected in this study. The serum osmolarity was calculated synchronously according to the serum concentrations of Na^+^, K^+^, glucose, and urea nitrogen.

**Results:**

In the present study, a significant difference was observed between the 28-day mortality of septic shock patients with hypo-osmolarity, hyper-osmolarity, and normal osmolarity (30.8%, 34.9%, and 23.0%, respectively, *p* < 0.001), which were detected at ICU admission. After propensity score matching (PSM) for basic characteristics, the relatively higher mortality was still observed in the hypo-osmolarity and hyper-osmolarity groups, compared to normal osmolarity group (30.6%, 30.0% vs. 21.7%, *p* = 0.009). Furthermore, we found that transforming the hyper-osmolarity into normal osmolarity by fluid therapy on day 2 and 3 decreased this mortality.

**Conclusion:**

The serum osmolarity disorder is markedly associated with increased 28-day mortality in septic shock patients.

## Introduction

Sepsis is a complex disorder in intensive care units (ICUs) and poses a severe health and economic burden on the patient and healthcare systems worldwide (1, 2). According to the latest definition (Sepsis-3), septic shock is defined as a subset of sepsis in which underlying circulatory, cellular and metabolic abnormalities are profound enough to substantially increase the risk of mortality (1–3). As concluded in a study by Vincent et.al. the frequency of septic shock for patients diagnosed at ICU admission was estimated to be 10.4% and the mean ICU mortality of septic shock patients was found to be 37.3% (3).

Over the past few decades, several risk factors, including serum albumin level, central venous pressure measurement, sequential organ failure assessment (SOFA) score and simplified acute physiology score II (SAPS II), have been identified to predict the mortality of septic shock patients in the ICU (4–7). However, the role of serum osmolarity at admission, which reflects the distribution of extracellular and intracellular water distribution, has not yet been studied in septic shock patients.

Serum osmolarity mainly depends on the concentrations of Na^+^, K^+^, glucose and urea nitrogen, and is strongly associated with the balance of various body fluids (8). As reported, serum Na^+^ disorder especially the hypernatremia has been studied to be an independent risk factor for ICU mortality (9–11). Also, the Na^+^ and K^+^ are important parts of acute physiology and chronic health evaluation II (APACHE II) score, which has been found to be independently associated with in-hospital mortality of sepsis patients (12). The relativity of serum osmolarity and disease severity or hospital mortality has already been studied in several patient populations, such as those with stroke, intracranial hemorrhage, acute coronary syndrome, and pulmonary disease and those admitted in the ICU (13–18). Despite the consistency of clinical results indicating that disorders in serum osmolarity are associated with increased hospital mortality, these conclusions are not applicable for septic shock patients.

In the present study, we performed a retrospective analysis investigating the relationship between admission serum osmolarity and ICU mortality in patients with septic shock. The essential information of the patients was extracted from the Medical Information Mart for Intensive Care IV (MIMIC-IV) database. Moreover, we studied whether the correction of serum osmolarity disorders after admission could have a positive effect on the outcome of these patients.

## Materials and methods

### Study population

This retrospective study collected data from the MIMIC-IV database (19). The establishment of this database was approved by the Massachusetts Institute of Technology (Cambridge, MA) and Beth Israel Deaconess Medical Center (Boston, MA); consent was obtained for the original data collection. Therefore, the ethical approval statement and the need for informed consent were waived for this manuscript. The permission of access to the database is obtained by author Gang H (Certification number 39516115). These patients diagnosed with septic shock were included in this study. Septic shock was defined according to the several criteria. Firstly, the SOFA score ≥ 2; secondly, consistent low blood pressure (MAP < 65 mmHg) was observed in which vasoactive drugs (such as norepinephrine, dopamine, dobutamine, and vasopressin) were utilized to maintain the pressure, despite adequate fluid resuscitation; thirdly, the lactate level > 2 mmol/L (20). We excluded patients who were younger than 18 years old, not firstly admitted in the ICU, the SOFA score < 2, duration of ICU stay < 24 h.

In our study, the data regarding the patients’ age, gender, weight, comorbidities, type of ICU admission, mean arterial pressure (MAP), heart rate, temperature, respiratory rate, oxygen saturation (SpO_2_), white blood cell (WBC) count, hemoglobin level, platelet (Plt) count, and sodium (Na^+^), potassium (K^+^), glucose, urea nitrogen, creatinine, albumin, and lactate levels detected at the admission to the ICU were included.

### Calculation of serum osmolarity

Serum osmolarity was calculated using the following equation (Na^+^ + K^+^) × 2 + (glucose/18) + (BUN/2.8). Only the values of each element measured at the same time were used in this study. In this study, 290–309 mmol/L was used as the normal range and reference group. Hypo-osmolarity was defined as the serum osmolarity at the first day was lower than 290 mmol/L; Hyper-osmolarity was defined as the serum osmolarity was higher than 309 mmol/L.

### Primary and secondary outcomes

The primary outcome of this study was the ICU 28-day mortality. The secondary outcomes included the hospital mortality, duration of ICU stay, volume of fluids administered, volume of urine output, blood product intake, corticoids intake, and acute kidney injury (AKI) incidence.

### Patient and public involvement

Neither the patients nor the public were involved in the design, planning, or reporting of this study.

### Statistical analysis

Continuous variables are presented as the mean and standard deviation (SD) or median and interquartile range (IQR). Categorical variables are presented as numbers and percentages. Comparisons between the groups were made using analysis of variance for continuous variables and χ^2^-test for categorical variables.

Multivariate logistic regression analysis was performed to characterize the relationship between serum osmolarity and ICU mortality. Baseline characteristics such as age, gender, weight, SOFA score, MAP, heart rate, hypertension, respiratory rate, temperature, SpO_2_, WBC, Plt counts, hemoglobin, creatinine, lactate levels, serum osmolarity group were selected to enter the multivariate logistic regression model. The missing values of variables were replaced using multivariate imputation by chained equations (MICE); subsequently, the values would be abandoned if the proportion of missing values was larger than 20%.

Except for multivariate logistic regression, propensity score matching (PSM) was used to adjust the covariates to ensure the robustness of our findings. For PSM, the basic admission characteristics including age, weight, gender, comorbidities, vital signs, and laboratory examination values were selected into the analysis, and 1:1 nearest neighbor matching with a caliper width of 0.05 was used. After PSM, standardized mean differences (SMDs) were used to evaluate the balance of values between two groups and the matched number for each group was 392. Afterward, logistic regression analysis was performed on the matched cohort. Results with *p*-values < 0.05 value were considered as statistically significant. All statistical analyses were performed in SPSS (version 25) and STATA (version 14).

## Results

### Baseline characteristics

**FIGURE 1:**
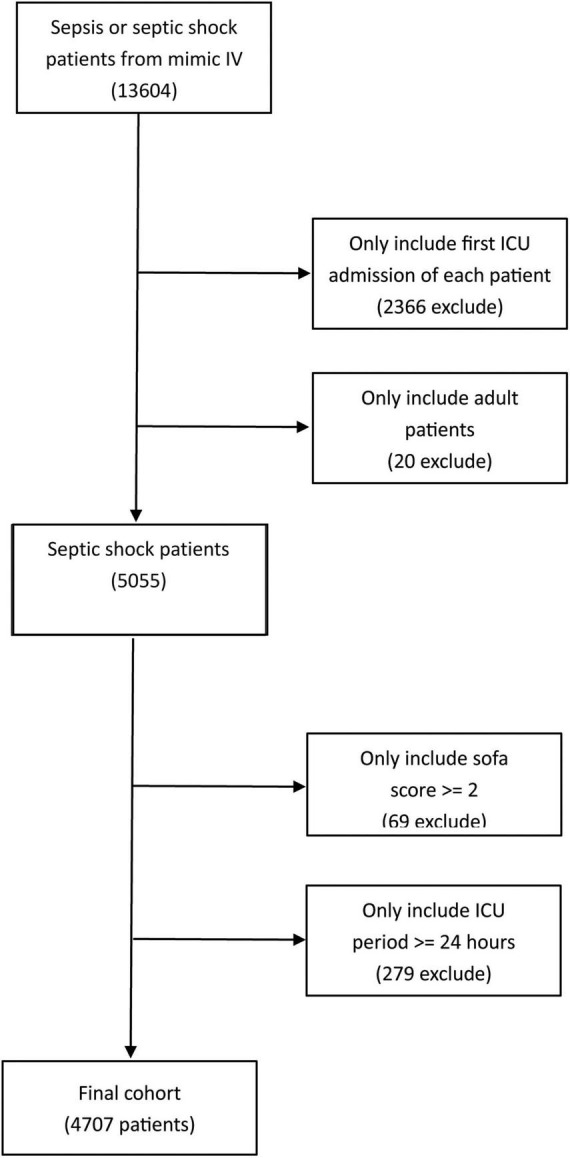
Flowchart of the cohort selection in the present study.

In the present study, the flowchart of the cohort selection is shown in Figure 1. Firstly, data for 13,604 patients diagnosed with sepsis from 2008 to 2019 were collected from this database. Then, we excluded patients who were not admitted into the ICU for the first time and those younger than 18 years old. Furthermore, we excluded sepsis patients without septic shock and 5,055 patients were preliminarily included in this study. Afterwards, according to the latest sepsis-3 criteria, 69 patients with a SOFA score lower than 2 points, and 279 patients with an ICU stay duration shorter than 24 h were excluded. Finally, 4,707 septic shock patients met the inclusion criteria and included in the final study.

The baseline characteristics of patients from each group are presented in Table 1. The patients in the hyper-osmolarity group were relatively older than those in the normal and hypo-osmolarity group (71.3 vs. 67.1 and 61.8 years, *p* = 0.001). Patients in the hyper-osmolarity group showed significantly higher sofa scores (10.4 vs. 8.7 and 8.8, *p* = 0.001) and lactate levels (4.6, 4.1, and 4.1 mmol/L, *p* = 0.001) than in the normal and hypo-osmolarity groups. In addition, the percentage of patients with a history of cardiovascular diseases (CAD), liver disease, renal disease, and diabetes were higher in the hyper-osmolarity group. Further, the vital signs and values of other laboratory tests at admission also show significant difference between three groups.

**TABLE 1:** Comparison of baseline characteristics between different serum osmolarity groups.

Variables	Serum osmolarity categories (mmol/L)			
	<290 (*n* = 633)	290–309 (*n* = 2,536)	>309 (*n* = 1,538)	*P*-value
Age (years)	61.8 ± 14.9	67.1 ± 15.9	71.3 ± 15.0	0.001
Male [n (%)]	320 (50.6%)	1,401 (55.2%)	881 (57.3%)	0.016
Weight (kg)	80.7 ± 23.1	84.2 ± 29.5	83.7 ± 26.9	0.019
SOFA score	8.7 ± 4.4	8.8 ± 4.0	10.4 ± 4.0	0.001
**Comorbidities**				
COPD [n (%)]	59 (9.3%)	269 (10.6%)	167 (10.9%)	0.556
CAD [n (%)]	95 (15.0%)	544 (21.5%)	402 (26.1%)	0.001
Hypertension [n (%)]	219 (34.6%)	951 (37.5%)	535 (34.8%)	0.143
Liver disease [n (%)]	125 (19.8%)	291 (11.5%)	138 (9.0%)	0.001
Renal disease [n (%)]	108 (17.1%)	760 (30.0%)	672 (43.7%)	0.001
Chronic thrombus [n (%)]	6 (1.0%)	18 (0.7%)	5 (0.3%)	0.163
Hyperlipidemia [n (%)]	206 (32.5%)	1,134 (44.7%)	708 (46.0%)	0.001
Diabetes [n (%)]	44 (7.0%)	233 (9.2%)	226 (14.7%)	0.001
Type of ICU				0.001
Medical [n (%)]	245 (38.7%)	816 (32.2%)	602 (39.1%)	
Surgical [n (%)]	83 (13.1%)	282 (11.1%)	154 (10.0%)	
Coronary [n (%)]	39 (6.2%)	263 (10.4%)	210 (13.7%)	
Cardiac [n (%)]	31 (4.9%)	233 (9.2%)	74 (4.8%)	
Trauma [n (%)]	54 (8.5%)	285 (11.2%)	134 (8.7%)	
**Vital signs**				
MAP (mmHg)	51.3 ± 13.0	51.9 ± 13.2	51.2 ± 13.7	0.381
Heart rate (/min)	94.3 ± 17.4	90.5 ± 17.2	89.1 ± 17.4	0.001
Respiratory rate (/min)	21.2 ± 4.4	21.0 ± 4.3	21.2 ± 4.3	0.244
Temperature (°C)	36.9 ± 0.7	36.9 ± 0.7	36.8 ± 0.8	0.001
SpO_2_ (%)	96.5 ± 2.3	96.6 ± 2.5	96.7 ± 3.1	0.001
Laboratory tests				
WBC (×10^9^/L)	15.2 ± 12.0	15.1 ± 11.9	15.7 ± 8.8	0.001
Hemoglobin (g/dL)	10.0 ± 1.9	10.6 ± 2.0	10.4 ± 2.1	0.001
Platelet (×10^9^/L)	188.9 ± 123.0	198.4 ± 116.6	203.0 ± 117.4	0.002
Creatinine (mg/dL)	1.3 ± 1.0	1.6 ± 1.3	2.6 ± 2.1	0.001
Lactate (mmol/L)	4.1 ± 3.0	4.1 ± 2.9	4.6 ± 3.4	0.001
Albumin (g/L)	2.9 ± 0.7	3.1 ± 0.7	3.0 ± 0.7	0.001

SOFA, Sequential Organ Failure Assessment; COPD, chronic obstructive pulmonary disease; CAD, cardiovascular diseases; MAP, mean arterial pressure; SpO_2_, oxygen saturation; WBC, white blood cell.

### Primary and secondary outcomes

The serum osmolarity was categorized into three groups and the outcomes within these groups were compared using the chi-square test or Kruskal-Wallis test. As shown in Table 2, the ICU 28-day mortality was significantly lower in the normal serum osmolarity group than in the hypo-osmolarity and hyper-osmolarity groups, respectively (23.0% vs. 30.8 and 34.9%; *p* = 0.001). This difference was also observed in hospital mortality of septic shock patients (*p* = 0.001). However, the durations of ICU stay (LOS) in the four groups did not show a significant difference (*p* = 0.561).

**TABLE 2:** Clinical outcomes by serum osmolarity categories.

Outcomes	Serum osmolarity categories (mmol/L)			
	<290 (*n* = 633)	290–309 (*n* = 2,536)	>309 (*n* = 1,538)	*P*-value
ICU mortality [n (%)]	195 (30.8%)	584 (23.0%)	536 (34.9%)	0.001
Hospital mortality [n (%)]	205 (32.4%)	629 (24.8%)	560 (36.4%)	0.001
ICU LOS [median (IQR)]	9.3 (4.9–17.9)	9.9 (5.7–18.0)	9.9 (5.2–17.8)	0.561
Albumin intake within 72 h [n (%)]	213 (33.6%)	626 (24.7%)	307 (20.0%)	0.001
Fluid intake in day 1 (mL)	9664.4 ± 8765.8	10128.4 ± 10149.3	11915.8 ± 21822.8	0.001
Fluid intake in day 2 (mL)	5375.3 ± 5502.6	5113.4 ± 4740.2	5580.7 ± 4828.9	0.005
Fluid intake in day 3 (mL)	4789.8 ± 4567.4	4448.9 ± 4132.3	4707.4 ± 4531.2	0.222
Urine output in day 1 (mL)	1766.5 ± 1448.8	1758.7 ± 1694.4	1429.5 ± 1529.6	0.001
Urine output in day 2 (mL)	1553.8 ± 1490.6	1603.5 ± 1885.5	1403.5 ± 1671.4	0.001
Urine output in day 3 (mL)	1653.1 ± 1412.0	1761.0 ± 1648.1	1597.0 ± 1627.2	0.002
Blood products within 72 h [n (%)]	396 (62.6%)	1,438 (56.7%)	825 (53.6%)	0.538
Corticoid use in ICU [n (%)]	125 (19.8%)	507 (20.0%)	328 (21.3%)	0.001
AKI incidence [n (%)]	343 (54.2%)	1,464 (57.7%)	985 (64.0%)	0.001

In the present study, we also compared the albumin intake, fluid intake, urine output, blood products intake, and corticoid intake in these groups; the detailed results are presented in Table 2. The patients from the hypo-osmolarity and normal osmolarity groups were administered less amounts of fluid (days 1 and 2; *p* < 0.01) and produced greater amounts of urine (days 1, 2, and 3; *p* < 0.01) than the patients from the hyper-osmolarity group. Moreover, the proportion of patients who received albumin was statistically higher in the hypo-osmolarity group (*p* = 0.001), while corticoids intake within the ICU duration was significantly higher in the hyper-osmolarity group (*p* = 0.001).

Furthermore, the PSM was used to balance the baseline characteristics between three groups. As shown in Table 3, there were 392 patients in each osmolarity group, the differences of clinical characteristics such as age, gender, sofa score, vital signs, and laboratory results between three groups were not statistically significant (*p* > 0.05). However, the ICU mortality and hospital mortality in the normal osmolarity group were significantly lower than that in the hypo-osmolarity and hyper-osmolarity group (*p* = 0.009 and *p* = 0.026, respectively).

**TABLE 3:** Comparison of clinical characteristics between different serum osmolarity groups after PSM.

Variables	Serum osmolarity categories (mmol/L)			
	<290 (*n* = 392)	290–309 (*n* = 392)	>309 (*n* = 392)	*P*-value
Age (years)	63.9 ± 14.1	64.3 ± 15.6	63.2 ± 16.2	0.559
Male [n (%)]	186 (47.5%)	178 (45.4%)	181 (46.2%)	0.846
SOFA score	9.0 ± 4.7	9.2 ± 4.2	9.5 ± 4.1	0.209
CAD [n (%)]	63 (16.1%)	61 (15.6%)	66 (16.8%)	0.888
Liver disease [n (%)]	62 (15.8%)	60 (15.3%)	62 (15.8%)	0.975
Renal disease [n (%)]	78 (19.9%)	80 (20.4%)	70 (17.8%)	0.633
Diabetes [n (%)]	30 (7.6%)	33 (8.4%)	31 (7.9%)	0.922
Heart rate (/min)	93.3 ± 17.7	92.2 ± 17.6	92.3 ± 17.0	0.633
Respiratory rate (/min)	21.2 ± 4.2	20.9 ± 4.3	21.3 ± 4.4	0.351
Temperature (°C)	36.9 ± 0.7	36.9 ± 0.7	36.9 ± 0.8	0.518
SpO_2_ (%)	96.5 ± 2.2	96.6 ± 2.6	96.6 ± 3.6	0.720
WBC (×10^9^/L)	15.3 ± 10.2	15.3 ± 11.0	15.6 ± 10.0	0.897
Hemoglobin (g/dL)	10.3 ± 1.9	10.3 ± 2.1	10.3 ± 2.1	0.856
Platelet (×10^9^/L)	189.0 ± 124.0	187.3 ± 118.0	194.6 ± 118.0	0.676
Creatinine (mg/dL)	1.4 ± 1.1	1.5 ± 0.9	1.6 ± 1.1	0.115
Lactate (mmol/L)	4.2 ± 3.0	4.2 ± 2.9	4.3 ± 3.1	0.823
Albumin (g/L)	3.0 ± 0.7	3.0 ± 0.7	3.0 ± 0.7	0.600
ICU mortality [n (%)]	120 (30.6%)	85 (21.7%)	116 (30.0%)	0.009
Hospital mortality [n (%)]	127 (32.4%)	95 (24.2%)	122 (31.2%)	0.026

### Hypo-osmolarity and hyper-osmolarity were independent risk factors for ICU mortality

Univariate and multivariate logistic regression analysis were used to identify the relationship between serum osmolarity and ICU mortality. As shown in Table 4, the result of multivariate logistic regression showed that both hypo-osmolarity and hyper-osmolarity were significantly associated with ICU mortality (OR = 1.301, *p* = 0.007 and OR = 1.470, *p* = 0.003; respectively). After PSM, the logistic regression was conducted again to study the risk factors for ICU mortality, and the results indicated that both hypo-osmolarity and hyper-osmolarity were independent risk factors (OR = 1.796, *p* = 0.002 and OR = 1.754, *p* = 0.006; respectively) (Table 5).

**TABLE 4:** Univariable and multivariable analysis of risk factors associated with ICU mortality before PSM.

Factors	Univariable analysis	Multivariable analysis		
	OR (95% CI)	*p*-value	OR (95% CI)	*p*-value
Age (years)	1.017 (1.013–1.021)	0.001	1.027 (1.021–1.034)	0.001
Male	1.084 (0.954–1.232)	0.216		
Sofa score	1.198 (1.178–2.218)	0.001	1.171 (1.142–1.200)	0.001
CAD	0.889 (0.761–1.039)	0.141		
Diabetes	0.686 (0.278–1.700)	0.425		
Liver disease	0.627 (0.500–0.787)	0.001	0.878 (0.680–1.133)	0.318
Renal disease	0.898 (0.783–1.030)	0.124		
Heart rate (/min)	1.013 (1.009–1.017)	0.001	1.012 (1.007–1.018)	0.001
Temperature (°C)	0.625 (0.569–0.687)	0.001	0.643 (0.562–0.735)	0.001
SpO_2_ (%)	0.880 (0.858–0.903)	0.001	0.891 (0.860–0.924)	0.001
WBC (×10^9^/L)	1.014 (1.008–1.021)	0.001	1.009 (1.000–1.017)	0.041
Hemoglobin (g/dL)	0.931 (0.901–0.961)	0.001	0.936 (0.896–0.978)	0.003
Platelet (×10^9^/L)	0.999 (0.998–1.000)	0.004	1.001 (1.000–1.002)	0.016
Creatinine (mg/dL)	1.125 (1.085–1.167)	0.001	1.001 (0.948–1.058)	0.965
Lactate (mmol/L)	1.192 (1.168–1.218)	0.001	1.112 (1.082–1.144)	0.001
Albumin (g/L)	0.561 (0.503–0.627)	0.001	0.745 (0.654–0.849)	0.001
Hyper-osmolarity (mmol/L)	1.488 (1.228–1.804)	0.001	1.470 (1.140–1.895)	0.003
Hypo-osmolarity (mmol/L)	1.788 (1.555–2.056)	0.001	1.301 (1.075–1.575)	0.007

**TABLE 5:** Univariable and multivariable analysis of risk factors associated with ICU mortality after PSM.

Factors	Univariable analysis	Multivariable analysis		
	OR (95% CI)	*p*-value	OR (95% CI)	*p*-value
Age (years)	1.013 (1.005–1.022)	0.002	1.027 (1.015–1.039)	0.001
Male	1.132 (0.876–1.464)	0.342		
Sofa score	1.203 (1.164–1.243)	<0.001	1.168 (1.113–1.225)	0.001
CAD	1.004 (0.761–1.423)	0.981		
Diabetes	0.523 (0.300–0.909)	0.022	0.572 (0.298–1.098)	0.093
Liver disease	1.355 (0.965–1.902)	0.079		
Renal disease	0.864 (0.619–1.203)	0.386		
Heart rate (/min)	1.013 (1.006–1.021)	0.001	1.014 (1.004–1.024)	0.006
Temperature (°C)	0.560 (0.465–0.676)	0.001	0.630 (0.493–0.806)	0.001
SpO_2_ (%)	0.856 (0.811–0.904)	0.001	0.885 (0.825–0.949)	0.001
WBC (×10^9^/L)	1.019 (1.008–1.032)	0.001	1.020 (1.006–1.034)	0.005
Hemoglobin (g/dL)	0.906 (0.849–0.967)	0.003	0.929 (0.856–1.009)	0.082
Platelet (×10^9^/L)	0.998 (0.997–0.999)	0.004	1.000 (0.999–1.002)	0.678
Creatinine (mg/dL)	1.306 (1.158–1.472)	0.001	1.036 (0.880–1.221)	0.669
Lactate (mmol/L)	1.217 (1.165–1.272)	0.001	1.117 (1.054–1.183)	0.001
Albumin (g/L)	0.540 (0.441–0.660)	0.001	0.844 (0.666–1.070)	0.162
Hyper-osmolarity (mmol/L)	1.518 (1.098–2.098)	0.011	1.754 (1.179–2.609)	0.006
Hypo-osmolarity (mmol/L)	1.593 (1.154–2.199)	0.005	1.796 (1.23–2.603)	0.002

### Normalization of serum osmolarity decreased the ICU mortality

**FIGURE 2:**
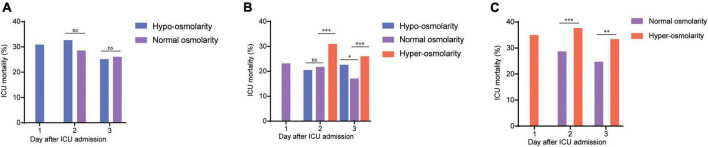
The dynamics of mortality and serum osmolarity within 3 days after ICU admission. **(A)** The dynamical ICU mortality of the patients with hypo-osmolarity at admission who transformed into the normal osmolarity state on days 2 and 3. **(B)** The dynamical ICU mortality of the patients with normal osmolarity at admission who transformed into the hypo-osmolarity or hyper-osmolarity states or maintained the normal osmolarity state on days 2 and 3. **(C)** The dynamical ICU mortality of the patients with hyper-osmolarity at admission who transformed into the normal osmolarity state on days 2 and 3. The changes in the mortality of patients in the hypo-osmolarity group who transformed into the hyper-osmolarity state and those in the hyper-osmolarity group who transformed into the hypo-osmolarity state are not presented as these results are in single digits; this could result in statistical bias. ^ns^*p* > 0.05; *p < 0.05; **p < 0.01; and ***p < 0.001.

In the present study, we also presented the dynamics of mortality and serum osmolarity within 3 days after ICU admission (Figure 2). As shown in Figure 2A, patients with hypo-osmolarity at admission transformed into those with normal osmolarity on days 2 and 3 after treatment did not show a significant decrease in mortality, compared with those that remained in the hypo-osmolarity state. However, this trend did not exist for patients from the normal osmolarity and hyper-osmolarity groups. In the normal osmolarity group, the ICU mortality of patients who maintained normal osmolarity or those that transformed into the hypo-osmolarity state was statistically lower than that of those that transformed into the hyper-osmolarity state on days 2 and 3 (*p* < 0.001) (Figure 2B). Obviously, in the hyper-osmolarity group, the patients who achieved normal osmolarity on days 2 and 3 showed a statistically lower ICU mortality (*p* < 0.001 and *p* < 0.01, respectively) than those who remained in the hyper-osmolarity state (Figure 2C).

## Discussion

Although many previous studies have studied the association between serum osmolarity and mortality in critically ill patients, emergency medical patients, and patients with stroke, intracranial hemorrhage, and acute coronary syndrome, only a few studies have identified the relationship between serum osmolarity and the ICU mortality of septic shock patients. In the present study, to the best of our knowledge, we demonstrated, for the first time, that serum osmolarity disorders were associated with significantly higher ICU 28-day mortality and higher hospital mortality than normal serum osmolarity. We also verified that the correction of serum osmolarity to the normal range was associated with a decreased mortality.

Fluid balance of the body is of vital importance for septic shock patients; serum osmolarity plays a significant role in the distribution of intracellular and extracellular fluid distribution (14, 21). As reported in a previous study, the perturbation of serum osmolarity is common in patients who are admitted to the ICU; this leads to the disturbance of body’s internal environment, potentially resulting in adverse outcomes (22). Hyper-osmolarity could lead to the mobilization of fluids from the venous capacitance vessels to the circulatory volume, thereby aggravating the hypoxia of organs or tissues (23, 24). Moreover, hyper-osmolarity is always accompanied by hypernatremia or hyperglycemia, which have been reported as separate risk factors for cardiac mortality (25, 26). A previous study reported that hypo-osmolarity at admission was also significantly associated with increased mortality in critically ill and emergency patients (22). However, the exact pathophysiological mechanism whereby hypo-osmolarity increases the mortality remains unknown. From our perspective, higher mortality at admission among patients from the hyper-osmolarity group may be associated with a higher rate of AKI at admission (Table 2), compared to the case for the patients from the hypo-osmolarity group. Besides, AKI leads to the decreased elimination of electrolytes and biochemical metabolites via urine, which in turn, aggravates the hyper-osmolarity (27).

For septic shock patients, hemodynamic instability is widely recognized as a risk factor for mortality, and many approaches have been used to detect and reverse this instability (28, 29). Blood pressure, MAP, and lactate concentration are three important factors indicating the fluid balance or tissue perfusion in the body; plenty of studies have focused on these parameters (30–32). Besides, clinical score systems, such as SOFA score, SAPS score, and APACHE score, have been studied in the diagnosis or the prognosis of the septic patients (6, 33). The score systems concentrate on the evaluation of the overall organs situation for the patients, and the fluid balance dynamics assessment is only a fraction of them, which might lead to significant bias using these score systems to reflect fluid instability. Therefore, it is important to find a parameter to indicate the fluid balance and predict the eventual outcome for septic shock patients. The serum osmolarity could simply reflect substantial organ dysfunction or the derangement of overall homeostatic mechanisms for salt, glucose, and urea in particular. Also, it is a very easy score to calculate and is based on the data that are generally available for most hospital admissions (22). Further, we identified that the treatment or normalization of the hyper-osmolarity detected at admission could lead to a reduction in mortality among septic shock patients, indicating that serum osmolarity could serve as a credible method to predict the ICU mortality of these patients.

Although this study is the first to investigate the relationship between serum osmolarity and the ICU mortality of patients diagnosed with septic shock and to study the effects of the normalization in serum osmolarity on ICU mortality, it still has several limitations. First, this is a retrospective study and could not strictly balance the baseline characteristics of patients in different serum osmolarity categories. Second, the serum osmolarity in the present study was calculated, rather than being measured directly; this could result in deviations from the actual serum osmolarity values despite the optimal calculation equation and simultaneous values of components being considered. Therefore, to further explore the relationship between serum osmolarity and the ICU mortality of septic shock patients, larger and prospective studies should be performed.

In conclusion, through the analysis of data from a large clinical database, our study indicates that both hypo-osmolarity and hyper-osmolarity at ICU admission were associated with increased mortality in patients with septic shock. Moreover, the normalization of hyper-osmolarity state may have positive effects in septic shock patients, i.e., decreased the mortality of these patients.

## Data availability statement

The original contributions presented in this study are included in the article/supplementary material, further inquiries can be directed to the corresponding authors.

## Ethics statement

The establishment of this database was approved by the Massachusetts Institute of Technology (Cambridge, MA) and Beth Israel Deaconess Medical Center (Boston, MA); consent was obtained for the original data collection. Therefore, the ethical approval statement and the need for informed consent were waived for this manuscript. Written informed consent to participate in this study was provided by the participants’ legal guardian/next of kin. Written informed consent was obtained from the individual(s) for the publication of any potentially identifiable images or data included in this article.

## Author contributions

JZ, TF, and WJ designed the study. GH, JZ, and YD performed the research and wrote the manuscript. BH, YS, JJ, DW, and ZL analyzed the data and conducted primary statistical analysis. All authors contributed to the article and approved the submitted version.
